# The PREEMPT study - evaluating smartphone-assisted n-of-1 trials in patients with chronic pain: study protocol for a randomized controlled trial

**DOI:** 10.1186/s13063-015-0590-8

**Published:** 2015-02-27

**Authors:** Colin Barr, Maria Marois, Ida Sim, Christopher H Schmid, Barth Wilsey, Deborah Ward, Naihua Duan, Ron D Hays, Joshua Selsky, Joseph Servadio, Marc Schwartz, Clyde Dsouza, Navjot Dhammi, Zachary Holt, Victor Baquero, Scott MacDonald, Anthony Jerant, Ron Sprinkle, Richard L Kravitz

**Affiliations:** Center for Health Care Policy and Research, University of California Davis, 2103 Stockton Blvd, Suite 2224, Sacramento, CA 95817 USA; Department of General Internal Medicine, University of California San Francisco, 1545 Divisadero St, Suite 308, San Francisco, CA 94118 USA; Open mHealth, 412 Broadway, Floor 2, New York, NY 10013 USA; Department of Biostatistics and Center for Evidence Based Medicine, Brown University School of Public Health, 121 South Main Street, Providence, RI 02912 USA; Department of Physical Medicine & Rehabilitation, 4150 V Street, Suite 1200, Sacramento, CA 95817 USA; VA Northern California Health Care System, 10535 Hospital Way, Mather, CA 95655 USA; Betty Irene Moore School of Nursing, University of California Davis, 4610 X Street, Sacramento, CA 95817 USA; Department of Psychiatry, Columbia University, 1051 Riverside Drive, Unit 48, New York, NY 10032 USA; Division of General Internal Medicine & Health Services Research, Department of Medicine, University of California Los Angeles, 911 Broxton Avenue, Los Angeles, CA 90024 USA; Cornell Tech, 111 8th Avenue, Number 302, New York, NY 10011 USA; 2Know, Inc, 257 Gold Street #1408, Brooklyn, NY 11201 USA; Department of Internal Medicine, University of California Davis, 4150 V Street, Suite 3100, Sacramento, CA 95817 USA; University of California Davis Medical Group - Folsom, 1370 Prairie City Road, Folsom, CA 95630 USA; University of California Davis Medical Group - Sacramento, 2825 J Street, Suite 300, Sacramento, CA 95816 USA; Department of Family and Community Medicine, University of California Davis, 4860 Y Street, Suite 2300, Sacramento, CA 95817 USA; University of California Davis Medical Group - Davis, 2660 West Covell Boulevard, Suites A, B and C, Davis, CA 95616 USA

**Keywords:** N-of-1, Chronic pain, eHealth, mHealth, Smartphone, Randomized controlled trial

## Abstract

**Background:**

Chronic pain is prevalent, costly, and clinically vexatious. Clinicians typically use a trial-and-error approach to treatment selection. Repeated crossover trials in a single patient (n-of-1 trials) may provide greater therapeutic precision. N-of-1 trials are the most direct way to estimate individual treatment effects and are useful in comparing the effectiveness and toxicity of different analgesic regimens. The goal of the PREEMPT study is to test the ‘Trialist’ mobile health smartphone app, which has been developed to make n-of-1 trials easier to accomplish, and to provide patients and clinicians with tools for individualizing treatments for chronic pain.

**Methods/design:**

A randomized controlled trial is being conducted to test the feasibility and effectiveness of the Trialist app. A total of 244 participants will be randomized to either the Trialist app intervention group (122 patients) or a usual care control group (122 patients). Patients assigned to the Trialist app will work with their clinicians to set up an n-of-1 trial comparing two pain regimens, selected from a menu of flexible options. The Trialist app provides treatment reminders and collects data entered daily by the patient on pain levels and treatment side effects. Upon completion of the n-of-1 trial, patients review results with their clinicians and develop a long-term treatment plan. The primary study outcome (comparing Trialist to usual care patients) is pain-related interference with daily functioning at 26 weeks.

**Discussion:**

Trialist will allow patients and clinicians to conduct personalized n-of-1 trials. In prior studies, n-of-1 trials have been shown to encourage greater patient involvement with care, which has in turn been associated with better health outcomes. mHealth technology implemented using smartphones may offer an efficient means of facilitating n-of-1 trials so that more patients can benefit from this approach.

**Trial registration:**

ClinicalTrials.gov: NCT02116621, first registered 15 April 2014.

**Electronic supplementary material:**

The online version of this article (doi:10.1186/s13063-015-0590-8) contains supplementary material, which is available to authorized users.

## Background

Chronic pain is highly prevalent [1] costly [2], and clinically vexatious [3]. Twenty percent of primary care patients are estimated to have persistent pain [4]. Patients experiencing persistent or chronic pain are more likely to have an anxiety or depressive disorder; they also are more likely to have limitations in physical functioning than patients without pain [4,5]. Pain is estimated to cost the United States (US) economy $560 to 630 billion annually due to health care expenditures and lost productivity [2]. Musculoskeletal pain is the most common reason for work disability and work absence [6].

Drug therapy is a mainstay of chronic pain management in primary care. Current drug treatment strategies for chronic painful conditions convey a mix of benefits and hazards. In usual practice, clinicians often begin with acetaminophen or a non-steroidal anti-inflammatory drug (NSAID), prescribing opioids when pain is severe or unresponsive [7,8]. When the analgesic response to the initial treatment is inadequate, clinicians can invoke stepped care, dose titration, opioid rotation, or augmentation with adjuvants such as anti-convulsants [8-10]. These approaches are usually employed in a non-systematic, trial-and-error fashion [11], which can appear to work in the short run but may lead to poor therapeutic decisions in the long run. A treatment that appears effective over a short period may only seem so because of random fluctuation in the patient’s underlying condition, uncontrollable external factors, placebo effect, or regression to the mean [7,12].

N-of-1 trials are single-subject crossover experiments [13] in which a patient completes repeated treatments comparing two treatment regimens. Also called single-patient trials [14], single-subject trials [15], single-case experiments [16], and individual-patient trials [14], n-of-1 trials switch patients back and forth between two treatments several times. Clinicians can then identify the more effective approach for an individual patient [12]. N-of-1 trials are appropriate for chronic, stable conditions and for treatments that have a rapid onset [14] and short half-life [15]. They are particularly suitable when available therapies are thought to have substantial heterogeneity of treatment effects (HTE), implying significant variation across patients as to which treatment works best. When HTE is large, average effects may mislead, calling for a more personalized approach [17].

N-of-1 trials are the most direct way to estimate individual treatment effects [14]. However, n-of-1 trials have not yet gained traction with clinicians, patients, and the scientific community. A major barrier is the perception that such trials demand too much time and effort [18]. The use of mobile health (mHealth) technologies to enhance care access and delivery [19] is a promising approach to reduce perceived barriers to n-of-1 participation. Smartphones are increasingly used in care innovation research [20] and provide an opportunity to develop interventions at lower cost and with decreased provider burden [21] than was possible before the integration of mobile technologies into daily life. Smartphones have been used to improve pain and health outcomes through the use of specialized software applications (apps) for assessing symptoms, facilitating communication between patients and providers, tracking outcomes [22], delivering information [23] and tracking behaviors. In pain settings, apps have been developed to record diary entries [24-26] and allow therapists to send tailored text messages to patients [26]. In non-pain settings, smartphones have been used in randomized controlled trials (RCTs) to track physical activity [27], monitor weight loss [21], and improve nutrition [28]. More than 125 million people in the US own smartphones, 50 million people own tablets [29], and smartphones account for more than 50% of mobile phone sales. Android (for example, Google Nexus series (Google, Mountain View, CA, USA), Samsung Galaxy series (Samsung, San Jose, CA, USA)) and iOS (for example, iPhone, iPad, iPod Touch (Apple Inc., Cupertino, CA, USA)) devices account for over 90% of the smartphone market [29].

N-of-1 trials have the potential to expand patient involvement and promote more personalized, patient-centered health care. From the population perspective, if mHealth-based n-of-1 trials can help patients and clinicians achieve therapeutic success faster and with greater confidence, patients may require fewer subsequent office visits, tests, emergency room visits, and after-hours telephone support, thus lessening the burden on health systems and saving money.

The goal of the PREEMPT (Personalized REsEarch for Monitoring Pain Treatment) study is to make n-of-1 trials easier to accomplish, to provide patients and clinicians with tools for individualizing treatments for chronic pain, and to evaluate this approach in terms of patient outcomes. A smartphone app called the ‘Trialist’ has been developed in collaboration with Open mHealth, a non-profit mobile health developer. The feasibility and efficacy of the ‘Trialist’ smartphone app is being assessed in a RCT to compare the effects of participating in a mobile n-of-1 trial versus usual care on patient outcomes including pain-related interference with daily functioning, pain intensity, participatory decision-making, medication adherence, and general health-related quality of life. Achieving these aims will set the stage for broader uptake of mHealth n-of-1 trials in chronic pain as well as other chronic health conditions.

## Methods/design

PREEMPT is a RCT with a planned total of 244 participants randomized to the Trialist app intervention or a usual care control group.

### Study setting

The study is located in Northern California with recruitment occurring within the University of California, Davis (UC Davis) Primary Care Network, UC Davis Family Medicine Clinic, UC Davis General Internal Medicine Clinic, and the Veterans Affairs Northern California Health Care System (VANCHS). These networks are located within the greater metropolitan areas of Sacramento and Yolo counties.

### Study hypothesis

The primary study hypothesis is that, compared to usual care, patients randomized to the Trialist will experience less pain interference (impairment of daily functioning including work outside the home, housework, and social activities) at 26 weeks follow-up. Secondary hypotheses are: compared to usual care, patients randomized to the Trialist will experience less pain interference, less pain intensity, better general health-related quality of life, improved participatory decision-making, greater satisfaction with pain treatment, better adherence to prescribed therapy, and a better patient experience with care, each measured as longitudinal change from baseline up to 52-weeks follow-up.

### Eligibility criteria

Study participants include patients as well as their regular treating clinicians. Clinicians are recruited first, and must have completed residencies in internal medicine, family medicine, or pain medicine or be practicing nurse practitioners or physician assistants. Patients, recruited from the practices of consenting clinicians, are required to meet the following criteria: English speaking adults between 18 and 75 years old who have experienced ongoing musculoskeletal pain for 6 weeks or longer; own an eligible iOS or Android smartphone or tablet; have a pain score of 4 or higher (on a 0 to 10 scale where 10 is the ‘worst pain imaginable’) on at least 1 of 3 items from the PEG pain scale [30,31]; and in the judgment of the treating clinician, have pain potentially amenable to treatment with acetaminophen, NSAIDs, low-dose opioids, tramadol, a complementary/alternative treatment such as massage or meditation, or a combination of these treatments (since these treatments are among those offered on the Trialist ‘menu’). Patients are excluded if they are pregnant or breastfeeding; have undergone surgery, radiation or chemotherapy treatment for cancer in the past 5 years; or have other medical conditions or behaviors, such as bipolar disorder or current alcohol or prescription drug abuse, rendering them unsuitable for the trial. (See Table 1 for a complete list of patient inclusion and exclusion criteria.)

**Table 1:** **Patient inclusion and exclusion criteria**

**Inclusion criteria**	**Exclusion criteria**
Experienced musculoskeletal pain for 6 weeks or longer	Currently pregnant or breastfeeding
Between 18 and 75 years old	Received surgery, radiation or chemotherapy treatment for cancer in the past 5 years
Android or iOS smartphone or tablet with a data plan and/or connected to a home WiFi network	Patient has a medical condition/s that would limit the patient life expectancy to < 2 years or imperil patient safety
A score of 4 or greater for at least one question of the PEG pain scale [31]	Dementia, bipolar disorder, schizophrenia, or active suicidality
Based on clinician judgment the patient is amenable to treatment with acetaminophen, non-steroidal anti-inflammatory drugs, short-acting opioids, tramadol, a complementary/alternative treatment such as massage or meditation, or a combination of these treatments	Evidence of alcohol or prescription drug abuse, or have a history of disruptive behavior
Ability to speak and read English	Failed five or more analgesic medications because of lack of effectiveness or poor tolerance

### Recruitment

Clinicians are recruited via flyers, Emails, letters and presentations. Once clinicians indicate interest, informed consent is obtained detailing their responsibilities and soliciting their consent to have their patients recruited into the study. Clinicians receive a $100 gift card for each patient who is enrolled and guided through the study.

**Figure 1:**
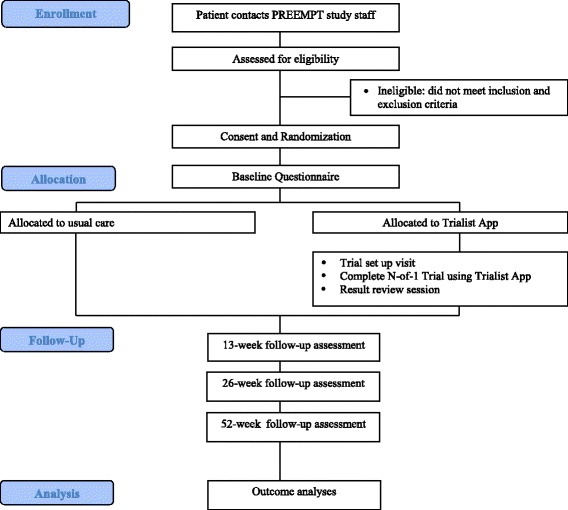
**Participant flow through recruitment process.**

Two methods are used for patient recruitment. First, clinicians can ask patients directly if they are interested in the study. Clinicians provide interested patients with a study flyer that provides research staff contact information. Second, patients of enrolled clinicians who have been seen within the past 2 to 12 months for a chronic painful condition (as indicated by appropriate International Statistical Classification of Diseases and Related Health Problems (ICD-9) codes) are sent an informational letter informing them about the study and inviting them to contact research staff if interested in learning more. Both patient recruitment methods rely on patients initiating contact with PREEMPT study research staff. (See Figure 1 for the participant flow diagram and Additional file 1 for the ICD-9 codes.) For completing the study, patients receive a gift card worth $50 (control patients) or $100 (intervention patients).

### Screening

Patients are screened for eligibility over the telephone. Research staff explain the study and ask initial screening questions to assess pain levels and determine that the patient has an eligible device. At this time, permission is obtained from the patient to contact his/her clinician for medical history screening. If permission is granted, the patient’s clinician is contacted via secure Email and/or telephone to verify that the patient is an appropriate candidate for the study. Eligible patients are then recontacted by telephone or Email, notified of eligibility, and asked the date and time of their next clinic appointment. Once a patient is deemed eligible, a consent packet is mailed or Emailed with the study consent form and Health Insurance Portability and Accountability Act (HIPAA) authorization form. Informed consent will be obtained from all participants included in this study.

### Randomization and allocation concealment

Patients are randomized to Trialist versus usual care. Randomization is stratified by clinician; each clinician’s patients are randomized in blocks of size 4 (90% of blocks) or 6 (10% of blocks) in order to balance the numbers of participants per clinician and to minimize selection bias. Patients assigned to usual care will receive the usual course of care as prescribed by their clinician. The allocation sequence are generated by the study statisticians (CS and JS) and provided to the study coordinator (MM) in a format that allows for clinician block size to be masked until the study is completed and for patient randomization allocation to be masked until completion of the enrollment procedures.

### Enrollment interview

The enrollment interview is conducted by research staff and occurs just prior to the patient’s outpatient appointment in the clinic waiting room. If the signed consent form and HIPAA authorization form (allowing the research team access to the patient’s medical records) have not been received prior to the enrollment interview, these documents are obtained first for each patient. Then the patient’s randomization assignment to either the Trialist app or usual care is revealed, and the patient completes a baseline questionnaire. At this visit, all patients receive a pain self-management booklet [32].

### Trialist intervention

Patients assigned to the intervention arm undergo a ‘Treatment Planning Visit’ with their clinician during a regularly scheduled appointment to design the patient’s n-of-1 trial. Clinicians and patients use the desktop interface of the Trialist together to select two treatment regimens for comparison. The customized option allows patients and clinicians to select from among acetaminophen; any NSAID (for example, ibuprofen, naproxen); an opioid combination product containing codeine, hydrocodone or oxycodone; tramadol; or complementary/alternative treatments such as massage, meditation or physical exercise. The participating provider’s clinical judgment and discussion with the patient determines which specific regimens to compare. Treatment regimens for comparison can be single agents (for example, acetaminophen) or combinations (for example, acetaminophen plus tramadol). Thus, the design of n-of-1 trials may range from simple (for example, acetaminophen versus low-dose hydrocodone/acetaminophen) to complex (low-dose acetaminophen/hydrocodone plus music therapy versus naproxen plus tramadol). If a clinician attempts to select combinations that are clinically inappropriate (for example, selecting two products both containing acetaminophen to be administered simultaneously), the Trialist will disallow that selection. The desktop interface also provides links to current prescribing standards and recommendations for the available drug treatment options. (See Additional file 2 for screenshots of the desktop interface.) Allowable n-of-1 trials will last a total of 4 to 12 weeks depending on the trial parameters selected. Trial parameters include the duration a patient is on each treatment before switching treatments (7 or 14 days), and the number of treatment pairs (cycles) they complete (2, 3, or 4). At least two cycles (for example, ABAB, BABA, ABBA, or BAAB) are required for a valid n-of-1 trial. (See Table 2 for examples of possible trial configurations.) The clinician and patient jointly select a start date for the n-of-1 trial, allowing for time to fill prescriptions. The n-of-1 trial parameter bounds were selected to provide a compromise between greater precision (for example, increasing number of cycles), and practicality (that is trial lengths that maintain patient interest).

**Table 2:** **Examples of potential treatment assignments with the Trialist**

**Period length (days)**	**Cycles**	**Trial duration (weeks)**	**Weeks**											
			**1**	**2**	**3**	**4**	**5**	**6**	**7**	**8**	**9**	**10**	**11**	**12**
7	2	4	A^a^	B^a^	B	A								
	3	6	A	B	B	A	A	B						
	4	8	B	A	B	A	A	B	B	A				
14	2	8	A		B		B		A					
	3	12	A		B		B		A		A		B	

^a^A and B are the alternative treatment regimens.

After an n-of-1 trial is set up, patients are provided login credentials for the Trialist smartphone app. The app is available free on Google Play and Apple’s App Store. Research staff provide patients with information on how to use the Trialist app, including a help guide and an online video tutorial (available on the study website, http://www.ucdmc.ucdavis.edu/chpr/preempt/). Research staff also provide patients with ongoing technical support.

**Figure 2:**
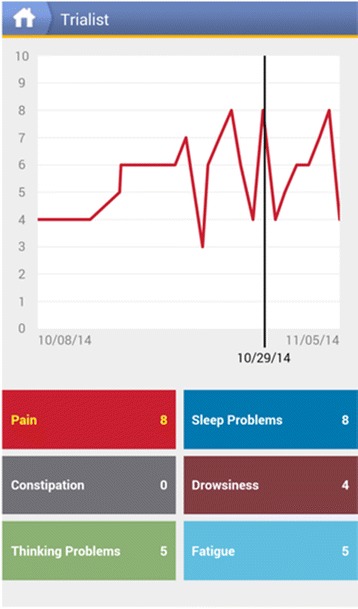
**Screenshot of the Trialist app showing a graph of a user’s responses to the daily questionnaire.**

**Figure 3:**
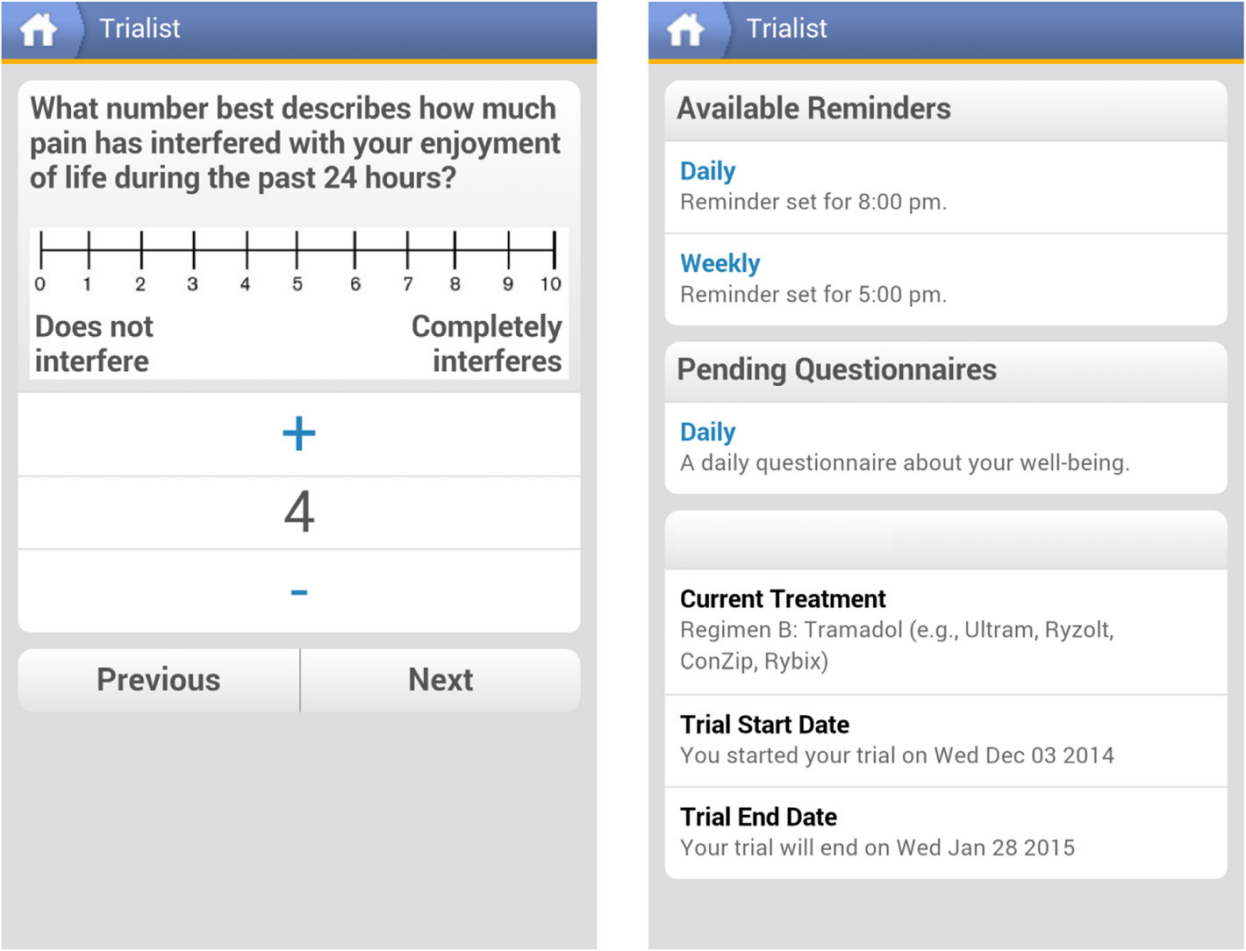
**Screenshots of the Trialist app showing a question from the daily questionnaire and the ‘Reminders’ screen.**

The Trialist app randomizes the treatment sequence and notifies the patients of the treatment they are scheduled to take, presents patients with a daily questionnaire tracking levels of pain and side effects of treatment such as fatigue and drowsiness, and provides daily reminders to complete the questionnaires. Most patients receive an 8-item daily questionnaire and 1 weekly question on adherence. Patients experiencing neuropathic pain can choose to specifically track 3 neuropathic symptoms (for a total of 11 daily items). Example daily questions include: ‘What number best describes your pain on average during the past 24 hours?’ on a 0 to 10 scale; ‘I felt fatigued during the past 24 hours’ on a 5-point scale from ‘not at all’ to ‘very much’; and ‘How often did you feel drowsy or sleepy today?’ on a 6-point response scale from ‘not at all’ to ‘very much’. The raw data are not included in the patient’s medical record, and are not available to other applications outside of the Trialist infrastructure. Patients receive notifications on their device to change treatments after 7 or 14 days and to complete daily and weekly questionnaires, and they also receive motivational messages keying off their progress in the trial. Patients can also view a graph of their own data to date, which displays scores from the questionnaires in chronological order (see Figure 2). Adherence to the daily questionnaires is essential to ensure successful completion of a patient’s n-of-1 trial. To improve adherence, patients are contacted by telephone and/or Email for: a) failure to start a trial by pressing the ‘start button’ in the Trialist app within 48 hours after a trial is due to start or b) completing fewer than 4 daily questionnaires in any week of the trial. All adherence-related support and contact with patients is recorded. See Figure 3 for screenshots of the Trialist app.

Upon completion of the n-of-1 trial, patients review trial results with their clinician during a ‘Results Review Session’. This visit occurs during a regularly scheduled office appointment. Clinician and patients will be advised to schedule a primary care appointment within 4 weeks of completing an n-of-1 trial, allowing treatment decisions to occur shortly after a trial is completed. Clinicians access the patient’s n-of-1 trial results using the Trialist desktop interface. The results are displayed in a series of graphs and text output. Six graphs will be displayed including raw data outputs and graphs showing probabilities of each treatment being more effective. (See Additional file 3 for the graphs available to a clinician and patient at the Result Review Session.)

### Patient measures

Patient measures are assessed at baseline, 13-, 26-, and 52 weeks through completion of an online or mailed questionnaire, with method of delivery based upon patient preference. Patient demographics, length of provider-patient relationship, and smartphone usage are measured at baseline. The domains measured at each time point include pain interference, pain intensity, self-reported adherence, participatory decision-making, satisfaction with pain treatment, trust in their clinician, and general health-related quality of life (HRQL). Study patients receive up to $50 (out of total incentive amount) for completing all the questionnaires.

#### Pain interference

Pain interference is assessed using the Patient Reported Outcomes Measurement Information System (PROMIS) adult Short Form v1.0 - Pain Interference 8a. The scale includes 8 items answered on a 5-point response scale (‘not at all’ to ‘very much’). Questions include: ‘How much did pain interfere with your day-to-day activities?’ and ‘How much did pain interfere with your family life?’ Further information on the PROMIS scales and measures are available from www.nihpromis.org.

#### Pain intensity

Pain intensity is assessed using the PROMIS adult scale v1.0 - Pain Intensity 3a. The scale includes 3 items on a 5-point response scale from ‘no pain’ to ‘very severe’. Questions include: ‘In the past 7 days, how intense was your pain at its worst?’ ‘In the past 7 days, how intense was your average pain?’ and ‘What is your level of pain right now?’

### Self-reported adherence

Self-reported adherence to pain treatment is assessed using the four-item Analgesic Adherence Scale developed to assess general medication adherence. The scale was developed by Rosser *et al*. [33], who replicated the work of McCracken *et al*. [34]. The scale comprises 4 items on a 5-point response scale (‘never’ to ‘always’). Questions include: ‘How often do you take less medication (smaller doses) than prescribed?’ and ‘How often do you miss a dose of medication?’.

#### Participatory decision-making

Four items drawn from the Consumer Assessment of Healthcare Providers and Systems (CAHPS) Patient-Centered Medical Home Survey assessing shared decision-making are included. The scale includes 2 items on a 4-point response scale (‘not at all’ to ‘a lot’), and 2 ‘yes/no’ questions. Questions include: ‘In the last 12 months, did you and this provider talk about starting or stopping a prescription medicine?’ and ‘When you talked about starting or stopping a prescription medicine, how much did this provider talk about the reasons you might want to take a medicine?’ [35-37].

#### Pain treatment satisfaction

Satisfaction with pain treatment is assessed using 22 items from the 61-item Pain Treatment Satisfaction Scale developed by Evans *et al*. [38]. Questions are asked on five-point response scales and assess how much information a patient would like to receive about their treatment, questions about a patient’s medical care (for example, ‘The medical staff is willing to provide me with the pain medication that I feel I need’), and questions about a patient’s current pain medications (for example, ‘My pain medication has a positive effect on my physical health’).

#### Patient-provider relationship

The patient-provider relationship is assessed using an adapted version of the 11-item Trust in Physician Scale developed by Thom *et al*. [39], where the term ‘provider’ is substituted for ‘doctor’. This allows the scale to be used with nurse practitioners and physician assistants. Questions are asked on a five-point response scale (‘totally agree’ to ‘totally disagree’). Questions include: ‘I trust my provider to put my medical needs above all other considerations when treating my medical problems’ and ‘My provider is well qualified to manage (diagnose and treat or make appropriate referral) medical problems like mine’.

#### General health-related quality of life (HRQL)

General HRQL is assessed using the 10-item PROMIS global health scale v.1.0/1.1 [40]. The scale includes 9 items on a 5-point response scale and one item on a 0 to 10 numerical scale. Questions include: ‘In general, how would you rate your physical health?’ and ‘How would you rate your pain on average?’

#### Demographics

Demographic data will be gathered on age, gender, marital status, race, ethnicity, employment and educational attainment. All demographic questions will be asked at baseline; marital status will be also re-assessed at 26 weeks, and employment status will be assessed at 13-, 26- and 52 weeks.

#### Smartphone usage

Smartphone usage will be assessed at baseline using a six-item scale to determine familiarity and frequency with smartphones and apps. Questions include: ‘How long have you been using a smartphone?’ (less than 6 months; between 6 months and 1 year; more than 1 year), and ‘Do you have any health-related Apps on your smartphone?’ (yes, no). Two questions are adapted from the smartphone and medical related app use questionnaire created by Payne [41].

#### Patient and clinician relationship length

Patient and clinician relationship length is assessed using 2 questions (items 3 and 4) from the 34-item CAHPS 12-month Clinician & Group Visit Survey. The questions assess how long the patient has been going to the provider and the number of visits to the provider in the last 12 months [42].

### Measures for intervention patients only

#### Patient trial expectations and experiences

Participants randomized to the Trialist app intervention group complete a Patient Expectations and Patient Experiences Questionnaire administered at the Treatment Planning Visit (pre-trial) and at the Result Review Visit (post-trial). The purpose of this questionnaire is to evaluate intervention patient expectations and experiences with treatment and the extent to which patient expectations were met. Questions are asked on a five-point response scale.

#### Trialist acceptability and satisfaction questionnaire

Intervention patients also complete the Trialist Acceptability and Satisfaction Questionnaire to provide feedback on the use of the Trialist app. The survey is sent to participants after the n-of-1 trial is completed. Questions contained in the survey are based upon adaptations of the System Usability Scale [43], and the Program Acceptability and Satisfaction Survey [44]. Questions are asked on five-point response scales (‘strongly disagree’ to ‘strongly agree’) and (‘not at all helpful’ to ‘extremely helpful’). Questions include: ‘I thought the Trialist app was easy to use’, ‘I found the Trialist app very awkward to use’, and ‘How satisfied were you with the reminders you received to complete your questionnaires?’

### Clinician questionnaire

At baseline, clinicians answer a 19-item questionnaire on their clinical specialty, clinical practice workload, clinical trial experience, smartphone usage and demographic characteristics. Questions include: ‘What is the average number of patients you see during a typical half-day of practice?’ and ‘During your training, residency or fellowship, how much clinical research experience did you have?’

### Sample size

The sample size required for the proposed RCT is based on the primary outcome: change from baseline to 26 weeks on the PROMIS pain interference scale. Assuming that the minimally important difference is 0.4 SD difference (4 points) and that 10% of those who enter the study will not complete an endpoint and will therefore be assigned a change of 0, the full sample (endpoint completers plus non-completers) would need to show a 3.6-point difference in order for the completers to show a minimally important difference. Assuming a common standard deviation of 10 points, each group (Trialist app and usual care) would need to include 122 patients (244 in total) in order to have 80% power to detect a 3.6-point difference in means using a 2-group *t*-test with a 0.05 2-sided significance level. Approximately 50 to 60 clinicians will be enrolled with each clinician being asked to enroll four to eight patients each. This reduces the burden required on any one clinician by ensuring that the maximum number of intervention patients for each clinician is two to four.

### Analytical plan

The primary analysis will be intent-to-treat which uses all participants as randomized. Outcomes will be analyzed both as changes from baseline to a single time point and as longitudinal evolutions in time. Changes at a single point (for example, from baseline to 26 weeks) between the groups will be compared by a *t*-test for continuous outcomes and chi-square test for binary outcomes. Longitudinal outcomes will use mixed models with a fixed effect of treatment and a random effect of time and a random time by treatment interaction using the appropriate generalized linear model link function and distribution (normal for continuous outcomes; binomial for binary ones; Poisson for counts). Additional exploratory analyses will examine: potential interactions of treatment with covariates such as age, gender, type of intervention, dosage, time on treatment, physician and clinic.

When no endpoint is available (for example, no pain measurements available at the 6-month time interval to calculate the outcome of change from baseline), we will use different approaches. In one, we will assume that no change has occurred and impute a change of zero. This will permit simple conservative assessments of single time point analyses. Longitudinal models can accommodate missing outcomes by ignoring them under the assumption that data are missing at random. We will also use multiple imputation to permit comprehensive analyses with missing covariates and interactions.

#### Analysis of N-of-1 trial results within the trialist (intervention group only)

As noted earlier, patients assigned to the Trialist are prompted to enter data on a daily basis. At the end of each person’s n-of-1 trial, statistical analysis is performed in order to compare results on the two treatments. Each n-of-1 trial requires a separate analysis and the analysis is automated to run in the background once each n-of-1 trial is completed. The analysis consists of running different Bayesian models that make different assumptions about the nature of the data (for example, data with and without correlation over time, with and without carryover across interventions, and so on). The results of these models are automatically compared as to which best fits the data, and the simplest model that accurately fits the data is chosen. The goal of the model checking is to assure that the model that can provide the most accurate and precise treatment effect is chosen. Automated model choice is checked manually by the study statisticians for all initial n-of-1 trials and then periodically thereafter to ensure that reasonable models are being selected. Robust models are preferred. Patients and clinicians are provided with an estimate of the treatment difference, represented as the estimated percentage that one treatment is superior to the other and a measure of its uncertainty (for example, 95% Bayesian confidence interval) as well as the probability that each treatment is the best for each outcome. Results are portrayed numerically and graphically (see Additional file 3.) Interpretation of results is left to the patient and clinician, but clinicians will have access to instructional materials on how to interpret the graphs generated by Trialist.

### Data management and monitoring

Outcome assessments will be collected via Research Electronic Data Capture (RedCap) survey or pen-and-paper. Data will be entered into RedCap databases [45]. All data that requires manual entry (for example, from a pen-and-paper surveys) will be entered by trained staff and undergo data quality and accuracy checks. Any data patients enter in the Trialist app is encrypted and uploaded to a secure server using Transport Layer Security (TLS)/Secure Sockets Layer (SSL) protocols [46,47]. A Safety Monitoring Committee (SMC) has been established. The SMC is an independent committee comprised of researchers and clinicians who are (with one exception) not involved in the study. SMC meetings are scheduled monthly, subject to cancellation at the discretion of the SMC chair provided there are no adverse events, no unanticipated problems, and no other issues for discussion. Unanticipated and adverse events will be reported to the SMC and to the institutional review board in accordance with University of California, Davis and Veterans Affairs Northern California Health Care System (VANCHS) procedures. The SMC will report adverse events considered related to the study directly to the National Institute of Nursing Research (NINR) program official.

### Ethics approval

Ethical approval was obtained from Institutional Review Boards at the University of California, Davis (496804) and the VANCHS (13-12-00717).

## Discussion

The Trialist app allows chronic pain patients and their clinicians to jointly set up and conduct personalized n-of-1 trials. Patients with chronic pain are at risk for both over-treatment and under-treatment. Thus the need for new approaches to chronic pain management is urgent, not least because of accumulating evidence that traditional approaches such as trial-and-error are often ineffective [11], prescription opioid overdoses are increasing, and prescription opioid abuse is a pressing clinical and economic problem [48].

For patients who are uncertain about which of two therapies to choose, or who have concerns about the relative benefits or about the side effects of various treatment options, or who have been on a treatment for a long time and simply do not know whether that treatment is working, n-of-1 trials can support more confident decision-making. N-of-1 trials encourage greater patient involvement with care, which has been associated with better health outcomes [49]. One reason nurses, physicians, and other practitioners ignore clinical evidence is that they question its relevance. N-of-1 data collected from their own patients or combined with similar data from other practices may have greater personal salience and more direct applicability to clinical care decisions [50].

N-of-1 trials have not gained traction in the research community, and one issue has been the difficulty of bringing n-of-1 trials to scale. The integration of smartphones and mHealth technology offers a possible solution, facilitating patient participation in n-of-1 trials and in turn, realizing benefit from increased therapeutic precision. With mHealth n-of-1 trials, clinical practice incorporates elements of research by bringing rigorous research design, outcomes assessment, and statistical analysis to the clinic. A successful demonstration of mHealth n-of-1 trials could pave the way for broader use of n-of-1 trials in chronic pain management and other chronic conditions.

## Trial status

At the time of manuscript submission 43 clinicians and 64 patients have been enrolled, and 23 patients have been randomized.

## Additional files

Additional file 1:
**ICD**-**9 Codes for chronic pain related diagnoses.**
Additional file 2:
**Trialist desktop interface.**
Additional file 3:
**Trialist graphs available at the Result Review Session.**

## Abbreviations

CAHPS: Consumer Assessment of Healthcare Providers and Systems
HIPPA: Health Insurance Portability and Accountability Act
HRQL: Health-related quality of life
HTE: Heterogeneity of treatment effects
ICD: International Statistical Classification of Diseases and Related Health Problems
NINR: National Institute of Nursing Research
NSAIDs: Non-steroidal anti-inflammatory drugs
PROMIS: Patient Reported Outcomes Measurement Information System
RCT: Randomized controlled trial
SMC: Safety Monitoring Committee
SSL: Secure Sockets Layer
TLS: Transport Layer Security
UC Davis: University of California Davis
US: United States
VANCHS: Veterans Affairs Northern California Health Care System
