# Supplementary material for: The PREEMPT study - evaluating smartphone-assisted n-of-1 trials in patients with chronic pain: study protocol for a randomized controlled trial
Source: Trials. 2015 Feb 27;16:67. doi: 10.1186/s13063-015-0590-8 (PMC4367894; doi:10.1186/s13063-015-0590-8)
Supplement: Additional file 1: — ICD-9 Codes for chronic pain related diagnoses. [file 13063_2015_590_MOESM1_ESM.pdf]

Additional File 1. ICD9 Codes for chronic pain related diagnoses

Dx Codes	Diagnosis Description	Dx Codes	Diagnosis Description
327.52	Sleep related leg cramps	717	Old bucket handle tear of medial meniscus
333.84	Organic writers' cramp	717.1	Derangement of anterior horn of medial meniscus
338	Central pain syndrome	717.2	Derangement of posterior horn of medial meniscus
338.21	Chronic pain due to trauma	717.3	Other and unspecified derangement of medial meniscus
338.28	Other chronic postoperative pain	717.4	Unspecified derangement of lateral meniscus
338.29	Other chronic pain	717.41	Bucket handle tear of lateral meniscus
338.4	Chronic pain syndrome	717.42	Derangement of anterior horn of lateral meniscus
710	Systemic lupus erythematosus	717.43	Derangement of posterior horn of lateral meniscus
711.08	Pyogenic arthritis, other specified sites	717.49	Other derangement of lateral meniscus
712.1	Chondrocalcinosis due to dicalcium phosphate crystals, unspecified site	717.5	Derangement of meniscus, not elsewhere classified
712.11	Chondrocalcinosis due to dicalcium phosphate crystals, shoulder region	717.6	Loose body in knee
712.12	Chondrocalcinosis due to dicalcium phosphate crystals, upper arm	717.7	Chondromalacia of patella
712.13	Chondrocalcinosis due to dicalcium phosphate crystals, forearm	717.81	Old disruption of lateral collateral ligament
712.14	Chondrocalcinosis due to dicalcium phosphate crystals, hand	717.82	Old disruption of medial collateral ligament
712.15	Chondrocalcinosis due to dicalcium phosphate crystals, pelvic region and thigh	717.83	Old disruption of anterior cruciate ligament
712.16	Chondrocalcinosis due to dicalcium phosphate crystals, lower leg	717.84	Old disruption of posterior cruciate ligament
712.17	Chondrocalcinosis due to dicalcium phosphate crystals, ankle and foot	717.85	Old disruption of other ligament of knee
712.18	Chondrocalcinosis due to dicalcium	717.89	Other internal derangement of knee

	phosphate crystals, other specified sites
712.19	Chondrocalcinosis due to dicalcium phosphate crystals, multiple sites
712.2	Chondrocalcinosis due to pyrophosphate crystals, unspecified site
712.21	Chondrocalcinosis due to pyrophosphate crystals, shoulder region
712.22	Chondrocalcinosis due to pyrophosphate crystals, upper arm
712.23	Chondrocalcinosis due to pyrophosphate crystals, forearm
712.24	Chondrocalcinosis due to pyrophosphate crystals, hand
712.25	Chondrocalcinosis due to pyrophosphate crystals, pelvic region and thigh
712.26	Chondrocalcinosis due to pyrophosphate crystals, lower leg
712.27	Chondrocalcinosis due to pyrophosphate crystals, ankle and foot
712.28	Chondrocalcinosis due to pyrophosphate crystals, other specified sites
712.29	Chondrocalcinosis due to pyrophosphate crystals, multiple sites
712.3	Chondrocalcinosis, cause unspecified, involving unspecified site
712.31	Chondrocalcinosis, cause unspecified, involving shoulder region
712.32	Chondrocalcinosis, cause unspecified, involving upper arm
712.33	Chondrocalcinosis, cause unspecified, involving forearm
717.9	Unspecified internal derangement of knee
718	Articular cartilage disorder, site unspecified
718.01	Articular cartilage disorder, shoulder region
718.02	Articular cartilage disorder, upper arm
718.03	Articular cartilage disorder, forearm
718.04	Articular cartilage disorder, hand
718.05	Articular cartilage disorder, pelvic region and thigh
718.07	Articular cartilage disorder, ankle and foot
718.08	Articular cartilage disorder, other specified site
718.09	Articular cartilage disorder, multiple sites
718.1	Loose body in joint, site unspecified
718.11	Loose body in shoulder joint
718.12	Loose body in upper arm joint
718.13	Loose body in forearm joint
718.14	Loose body in hand joint

712.34	Chondrocalcinosis, cause unspecified, involving hand	718.15	Loose body in pelvic joint
712.35	Chondrocalcinosis, cause unspecified, involving pelvic region and thigh	718.17	Loose body in ankle and foot joint
712.36	Chondrocalcinosis, cause unspecified, involving lower leg	718.18	Loose body in joint of other specified site
712.37	Chondrocalcinosis, cause unspecified, involving ankle and foot	718.19	Loose body in joint of multiple sites
712.38	Chondrocalcinosis, cause unspecified, involving other specified sites	719.4	Pain in joint, site unspecified
712.39	Chondrocalcinosis, cause unspecified, involving multiple sites	719.41	Pain in joint, shoulder region
712.8	Other specified crystal arthropathies, site unspecified	719.42	Pain in joint, upper arm
712.81	Other specified crystal arthropathies, shoulder region	719.43	Pain in joint, forearm
712.82	Other specified crystal arthropathies, upper arm	719.44	Pain in joint, hand
712.83	Other specified crystal arthropathies, forearm	719.45	Pain in joint, pelvic region and thigh
712.84	Other specified crystal arthropathies, hand	719.46	Pain in joint, lower leg
712.85	Other specified crystal arthropathies, pelvic region and thigh	719.47	Pain in joint, ankle and foot
712.86	Other specified crystal arthropathies, lower leg	719.48	Pain in joint, other specified sites
712.87	Other specified crystal arthropathies, ankle and foot	719.49	Pain in joint, multiple sites
712.88	Other specified crystal arthropathies, other specified sites	719.5	Stiffness of joint, not elsewhere classified, unspecified site
712.89	Other specified crystal arthropathies, multiple sites	719.51	Stiffness of joint, not elsewhere classified, shoulder region
712.9	Unspecified crystal arthropathy, site unspecified	719.52	Stiffness of joint, not elsewhere classified, upper arm
712.91	Unspecified crystal arthropathy, shoulder region	719.53	Stiffness of joint, not elsewhere classified, forearm

712.92	Unspecified crystal arthropathy, upper arm
712.93	Unspecified crystal arthropathy, forearm
712.94	Unspecified crystal arthropathy, hand
712.95	Unspecified crystal arthropathy, pelvic region and thigh
712.96	Unspecified crystal arthropathy, lower leg
712.97	Unspecified crystal arthropathy, ankle and foot
712.98	Unspecified crystal arthropathy, other specified sites
712.99	Unspecified crystal arthropathy, multiple sites
714	Rheumatoid arthritis
714.89	Other specified inflammatory polyarthropathies
715	Generalized osteoarthrosis, unspecified site
715.04	Generalized osteoarthrosis, involving hand
715.09	Generalized osteoarthrosis, involving multiple sites
715.1	Primary localized osteoarthrosis, specified site
715.11	Primary localized osteoarthrosis, shoulder region
715.12	Primary localized osteoarthrosis, upper arm
715.13	Primary localized osteoarthrosis, forearm
715.14	Primary localized osteoarthrosis, hand
715.15	Primary localized osteoarthrosis, pelvic region and thigh
715.16	Primary localized osteoarthrosis,
719.54	Stiffness of joint, not elsewhere classified, hand
719.55	Stiffness of joint, not elsewhere classified, pelvic region and thigh
719.56	Stiffness of joint, not elsewhere classified, lower leg
719.57	Stiffness of joint, not elsewhere classified, ankle and foot
719.58	Stiffness of joint, not elsewhere classified, other specified site
719.59	Stiffness of joints, not elsewhere classified, multiple sites
721	Cervical spondylosis without myelopathy
721.3	Lumbosacral spondylosis without myelopathy
721.7	Traumatic spondylopathy
721.8	Other allied disorders of spine
721.9	Spondylosis of unspecified site without mention of myelopathy
722.4	Degeneration of cervical intervertebral disc
722.51	Degeneration of thoracic or thoracolumbar intervertebral disc
722.52	Degeneration of lumbar or lumbosacral intervertebral disc
722.6	Degeneration of intervertebral disc, site unspecified
722.8	Postlaminectomy syndrome, unspecified region
722.81	Postlaminectomy syndrome, cervical region
722.82	Postlaminectomy syndrome, thoracic region
722.83	Postlaminectomy syndrome, lumbar region
723	Spinal stenosis in cervical region

	lower leg		
715.17	Primary localized osteoarthrosis, ankle and foot	723.1	Cervicalgia
715.18	Primary localized osteoarthrosis, other specified sites	723.2	Cervicocranial syndrome
715.2	Secondary localized osteoarthrosis, unspecified site	723.3	Cervicobrachial syndrome (diffuse)
715.21	Secondary localized osteoarthrosis, shoulder region	723.4	Brachial neuritis or radiculitis nos
715.22	Secondary localized osteoarthrosis, upper arm	724	Spinal stenosis, unspecified region other than cervical
715.23	Secondary localized osteoarthrosis, forearm	724.01	Spinal stenosis of thoracic region
715.24	Secondary localized osteoarthrosis, involving hand	724.02	Spinal stenosis, lumbar region, without neurogenic claudication
715.25	Secondary localized osteoarthrosis, pelvic region and thigh	724.03	Spinal stenosis, lumbar region, with neurogenic claudication
715.26	Secondary localized osteoarthrosis, lower leg	724.09	Spinal stenosis, other region other than cervical
715.27	Secondary localized osteoarthrosis, ankle and foot	724.1	Pain in thoracic spine
715.28	Secondary localized osteoarthrosis, other specified site	724.2	Lumbago
715.3	Localized osteoarthrosis not specified whether primary or secondary, unspecified site	724.3	Sciatica
715.31	Localized osteoarthrosis not specified whether primary or secondary, shoulder region	724.4	Thoracic or lumbosacral neuritis or radiculitis, unspecified
715.32	Localized osteoarthrosis not specified whether primary or secondary, upper arm	724.5	Unspecified backache
715.33	Localized osteoarthrosis not specified whether primary or secondary, forearm	726.1	Unspecified disorders of bursae and tendons in shoulder region
715.34	Localized osteoarthrosis not specified whether primary or secondary, hand	726.11	Calcifying tendinitis of shoulder
715.35	Localized osteoarthrosis not specified whether primary or secondary, pelvic	726.12	Bicipital tenosynovitis

	region and thigh		
715.36	Localized osteoarthrosis not specified whether primary or secondary, lower leg	726.31	Medial epicondylitis of elbow
715.37	Localized osteoarthrosis not specified whether primary or secondary, ankle and foot	726.32	Lateral epicondylitis of elbow
715.38	Localized osteoarthrosis not specified whether primary or secondary, other specified sites	726.33	Olecranon bursitis
715.8	Osteoarthrosis involving more than one site, but not specified as generalized, unspecified site	726.39	Other enthesopathy of elbow region
715.89	Osteoarthrosis involving multiple sites, but not specified as generalized	726.61	Pes anserinus tendinitis or bursitis
715.9	Osteoarthrosis, unspecified whether generalized or localized, unspecified site	726.62	Tibial collateral ligament bursitis
715.91	Osteoarthrosis, unspecified whether generalized or localized, shoulder region	726.63	Fibular collateral ligament bursitis
715.92	Osteoarthrosis, unspecified whether generalized or localized, upper arm	726.64	Patellar tendinitis
715.93	Osteoarthrosis, unspecified whether generalized or localized, forearm	726.65	Prepatellar bursitis
715.94	Osteoarthrosis, unspecified whether generalized or localized, hand	726.71	Achilles bursitis or tendinitis
715.95	Osteoarthrosis, unspecified whether generalized or localized, pelvic region and thigh	726.72	Tibialis tendinitis
715.96	Osteoarthrosis, unspecified whether generalized or localized, lower leg	727	Unspecified synovitis and tenosynovitis
715.97	Osteoarthrosis, unspecified whether generalized or localized, ankle and foot	727.01	Synovitis and tenosynovitis in diseases classified elsewhere
715.98	Osteoarthrosis, unspecified whether generalized or localized, other specified sites	727.04	Radial styloid tenosynovitis
716.1	Traumatic arthropathy, site unspecified	727.05	Other tenosynovitis of hand and wrist

716.11	Traumatic arthropathy, shoulder region	727.06	Tenosynovitis of foot and ankle
716.12	Traumatic arthropathy, upper arm	727.09	Other synovitis and tenosynovitis
716.13	Traumatic arthropathy, forearm	727.2	Specific bursitides often of occupational origin
716.14	Traumatic arthropathy, hand	727.3	Other bursitis disorders
716.15	Traumatic arthropathy, pelvic region and thigh	727.81	Contracture of tendon (sheath)
716.16	Traumatic arthropathy, lower leg	728.85	Spasm of muscle
716.17	Traumatic arthropathy, ankle and foot	728.89	Other disorder of muscle, ligament, and fascia
716.18	Traumatic arthropathy, other specified sites	728.9	Unspecified disorder of muscle, ligament, and fascia
716.19	Traumatic arthropathy, multiple sites	729.1	Unspecified myalgia and myositis
716.49	Transient arthropathy, multiple sites	729.2	Unspecified neuralgia, neuritis, and radiculitis
716.5	Unspecified polyarthropathy or polyarthritis, site unspecified	729.3	Panniculitis, unspecified site
716.51	Unspecified polyarthropathy or polyarthritis, shoulder region	729.39	Panniculitis of other sites
716.52	Unspecified polyarthropathy or polyarthritis, upper arm	729.5	Pain in soft tissues of limb
716.53	Unspecified polyarthropathy or polyarthritis, forearm	729.82	Cramp of limb
716.54	Unspecified polyarthropathy or polyarthritis, hand	729.89	Other musculoskeletal symptoms referable to limbs
716.55	Unspecified polyarthropathy or polyarthritis, pelvic region and thigh	780.96	Generalized pain
716.56	Unspecified polyarthropathy or polyarthritis, lower leg	784.92	Jaw pain
716.57	Unspecified polyarthropathy or polyarthritis, ankle and foot	786.5	Unspecified chest pain
716.58	Unspecified polyarthropathy or polyarthritis, other specified sites	786.51	Precordial pain
716.59	Unspecified polyarthropathy or polyarthritis, multiple sites	786.52	Painful respiration
