# Supplementary material for: The PREEMPT study - evaluating smartphone-assisted n-of-1 trials in patients with chronic pain: study protocol for a randomized controlled trial
Source: Trials. 2015 Feb 27;16:67. doi: 10.1186/s13063-015-0590-8 (PMC4367894; doi:10.1186/s13063-015-0590-8)
Supplement: Additional file 2: — Trialist desktop interface. [file 13063_2015_590_MOESM2_ESM.pdf]

Additional File 2. Trialist desktop interface

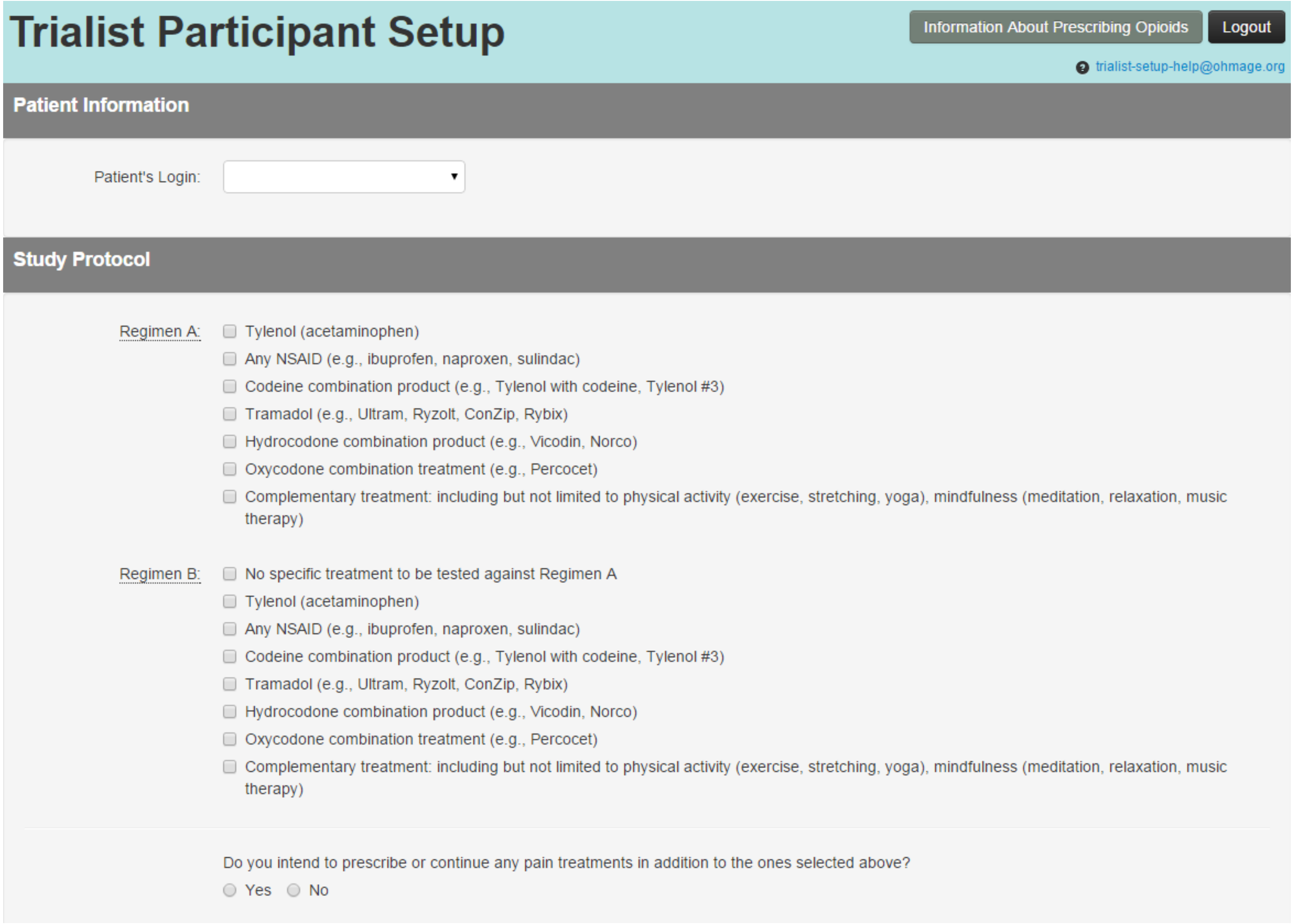

Trialist Participant Setup

Information About Prescribing Opioids | Logout

trialist-setup-help@ohmage.org

Patient Information

Patient's Login:

Study Protocol

Regimen A:

- Tylenol (acetaminophen)
- Any NSAID (e.g., ibuprofen, naproxen, sulindac)
- Codeine combination product (e.g., Tylenol with codeine, Tylenol #3)
- Tramadol (e.g., Ultram, Ryzolt, ConZip, Rybix)
- Hydrocodone combination product (e.g., Vicodin, Norco)
- Oxycodone combination treatment (e.g., Percocet)
- Complementary treatment: including but not limited to physical activity (exercise, stretching, yoga), mindfulness (meditation, relaxation, music therapy)

Regimen B:

- No specific treatment to be tested against Regimen A
- Tylenol (acetaminophen)
- Any NSAID (e.g., ibuprofen, naproxen, sulindac)
- Codeine combination product (e.g., Tylenol with codeine, Tylenol #3)
- Tramadol (e.g., Ultram, Ryzolt, ConZip, Rybix)
- Hydrocodone combination product (e.g., Vicodin, Norco)
- Oxycodone combination treatment (e.g., Percocet)
- Complementary treatment: including but not limited to physical activity (exercise, stretching, yoga), mindfulness (meditation, relaxation, music therapy)

Do you intend to prescribe or continue any pain treatments in addition to the ones selected above?

Yes No

Study Start Date:

Period Length:

Number of Cycles:

Mandatory Outcomes

These are outcomes that will be tracked by every participant in the study.

- A daily questionnaire about pain intensity and interference.
- A daily question about how much fatigue you felt in the past day.
- A daily question about how drowsy you felt during the current day.
- A daily question about problems moving your bowels.
- A daily question about the previous night's sleep.

Pain medicines can sometimes affect our thinking or concentration. Please select one of the following questions about your thinking or concentration to track every day.

Optional Neuropathic Pain Outcome

Does the patient have pain accompanied by burning, pins and needles, itching, or a sensitive or unpleasant sensation on the skin?

Yes No

Create and Assign Protocol
