# Supplementary material for: The PREEMPT study - evaluating smartphone-assisted n-of-1 trials in patients with chronic pain: study protocol for a randomized controlled trial
Source: Trials. 2015 Feb 27;16:67. doi: 10.1186/s13063-015-0590-8 (PMC4367894; doi:10.1186/s13063-015-0590-8)
Supplement: Additional file 3: — Trialist graphs available at the Result Review Session. [file 13063_2015_590_MOESM3_ESM.pdf]

Additional File 3. Trialist graphs available at the Result Review Session

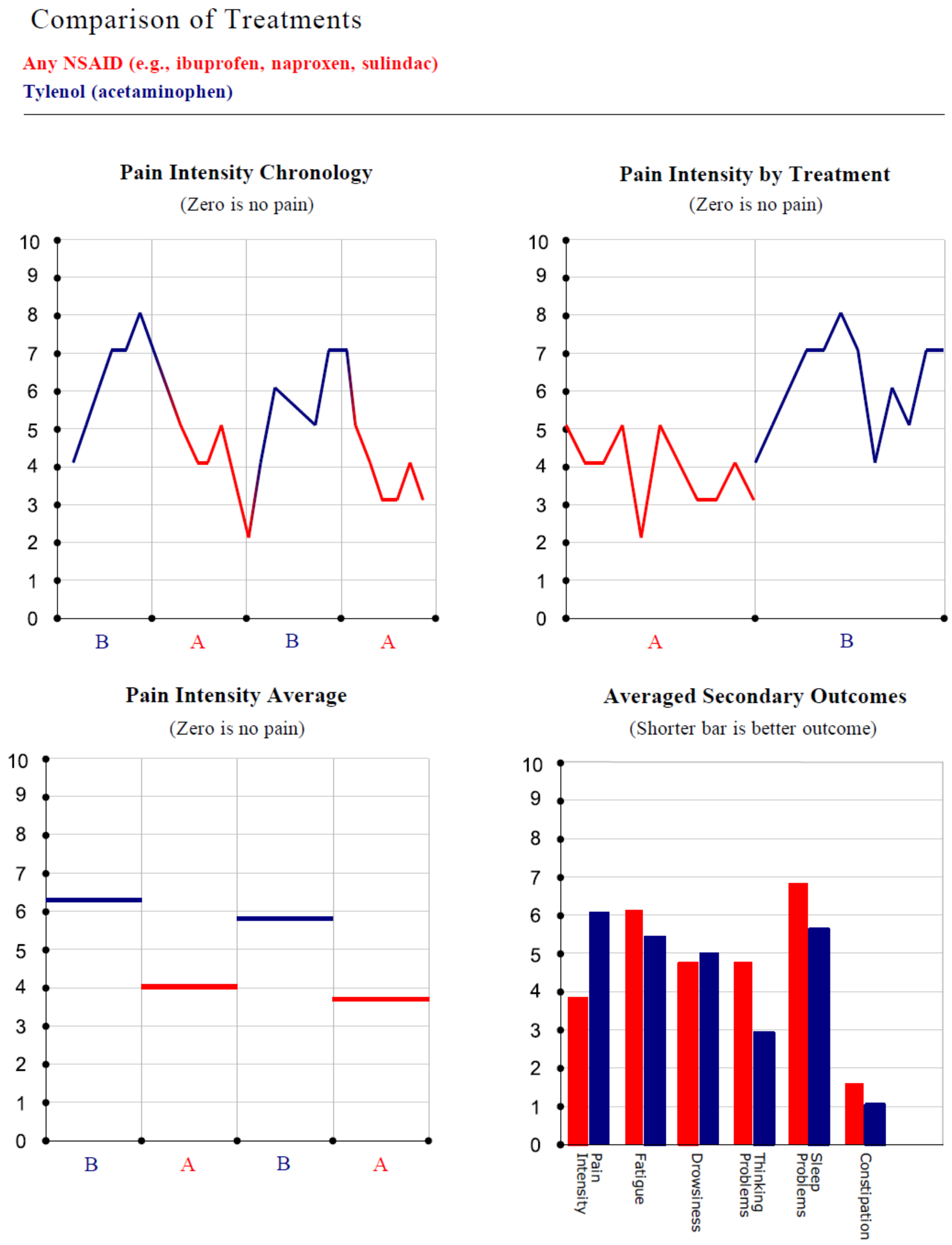

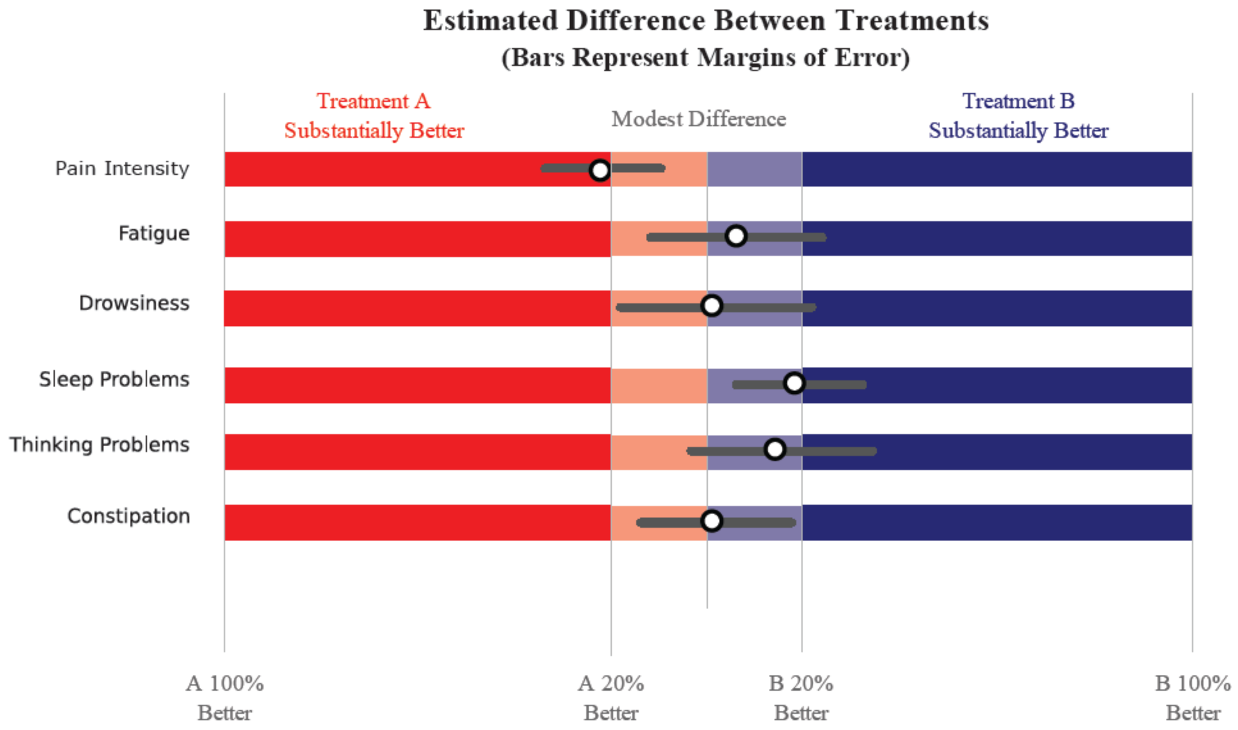
Estimated Difference Between Treatments
(Bars Represent Margins of Error)
Treatment A
Substantially Better
Modest Difference
Treatment B
Substantially Better
Pain Intensity
Fatigue
Drowsiness
Sleep Problems
Thinking Problems
Constipation
A 100%
Better
A 20%
Better
B 20%
Better
B 100%
Better

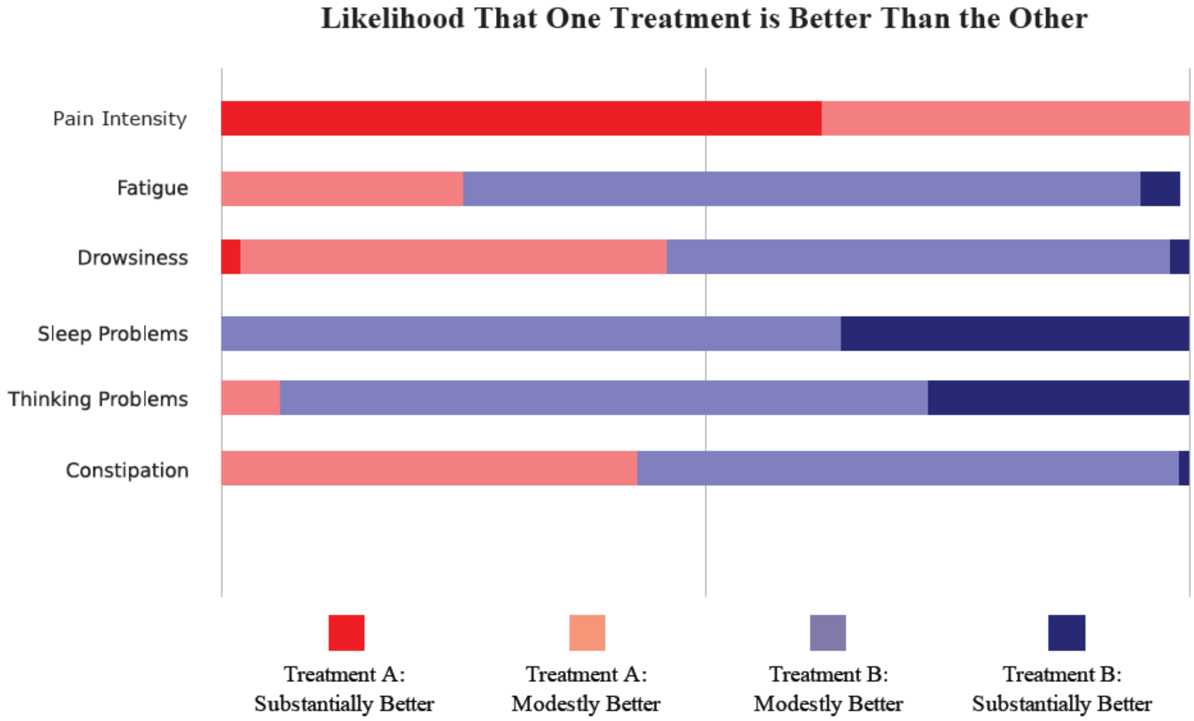
Likelihood That One Treatment is Better Than the Other
Pain Intensity
Fatigue
Drowsiness
Sleep Problems
Thinking Problems
Constipation
Treatment A:
Substantially Better
Treatment A:
Modestly Better
Treatment B:
Modestly Better
Treatment B:
Substantially Better
